# Healthcare Experiences of Older Adults with an LGBT+ Identity: An Integrative Review

**DOI:** 10.3390/healthcare14081110

**Published:** 2026-04-21

**Authors:** Anders Valentin Johansen, Christine Elise Swane, Lotte Evron, Laila Twisttmann Bay, Sinthuja Vasantharajan, Dorthe Susanne Nielsen

**Affiliations:** 1 Department of Geriatric Medicine, Odense University Hospital, 5000 Odense, Denmark; 2 Department of Infectious Diseases, Odense University Hospital, 5000 Odense, Denmark; 3 COPE Research Center, University of Southern Denmark, 5230 Odense, Denmark; dorthe.nielsen@rsyd.dk; 4 EGV Foundation—Social Inclusion of Older Adults, 1361 Copenhagen, Denmark; csw@egv.dk; 5 Ingeborggaarden Nursing Home, Frederiksberg Municipality, 2000 Frederiksberg, Denmark; lotevr@frederiksberg.dk; 6 Department of Gynaecology, Odense University Hospital, 5000 Odense, Denmark; laila.t.bay@rsyd.dk; 7 Department of Occupational Therapy and Nursing, University College Lillebaelt, 5230 Odense, Denmark; sivi@ucl.dk; 8 Geriatric Research Department, Odense University Hospitals, 5000 Odense, Denmark

**Keywords:** older, LGBT+, healthcare, integrative review, vulnerability

## Abstract

**Background/Objectives:** Older adults with an LGBT+ identity represent an under-researched population within healthcare systems. Existing evidence suggests that they experience distinct health challenges compared to their heterosexual counterparts, partly shaped by lifelong experiences of stigma and discrimination. Such experiences may contribute to minority stress, which is associated with adverse mental health outcomes and lifestyle-related health issues. This review aims to synthesise the existing literature on how older adults with an LGBT+ identity experience encounters with healthcare. **Methods**: An integrative literature review was conducted following PRISMA guidelines. A systematic search of multiple databases was performed, and studies were screened using predefined inclusion and exclusion criteria. Data were analysed using systematic text condensation. **Results:** A total of 18 studies were included, comprising approximately 450 participants. All studies contained a qualitative component. Three overarching themes were identified: (1) double-edged discrimination—experiences of stigma and anticipated fear in healthcare; (2) relational networks as essential yet fragile sources of support in later life; and (3) healthcare practices as shaping inclusion or invisibility—the need for competence and recognition. **Conclusions:** The findings highlight significant barriers faced by older adults with an LGBT+ identity in healthcare, including fear of discrimination and challenges related to disclosure. Social networks play a crucial role as sources of support, while healthcare professionals’ competencies and practices are central to ensuring inclusive and equitable care.

## 1. Introduction

The global population is ageing rapidly. The United Nations estimates that by 2050, the number of people aged 60 years and older will be twice that of children under the age of five [1,2]. This demographic shift presents significant challenges, including increasing demands on health and social care systems, particularly in relation to long-term care [3].

Research indicates that older adults with an LGBT+ identity are often affected by past experiences of discrimination in healthcare, contributing to increased vulnerability both within healthcare systems and in society more broadly [4,5]. Such experiences may limit access to healthcare services. Reported forms of discrimination include refusal of treatment, lack of recognition of same-sex relationships, heteronormative communication, and situations where individuals feel compelled to disclose their sexual identity [6]. These experiences are often described as stressful and may discourage openness due to fear of discrimination [7,8]. Previous research has also highlighted challenges in healthcare encounters. Reviews indicate that older adults with an LGBT+ identity may face prejudice related to caregivers’ personal or religious beliefs, as well as assumptions about LGBT+ lifestyles [9,10,11]. Such assumptions—for example, regarding promiscuity—may influence how individuals are treated and may affect their willingness to seek healthcare.

Life-course experiences play an important role in shaping health outcomes. Older adults with an LGBT+ identity have often been exposed to stigma over extended periods, contributing to what is described as minority stress [12,13,14]. This chronic stress may increase the risk of mental health challenges, cognitive decline, and lifestyle-related health conditions [15]. Higher prevalence of smoking, alcohol use, substance abuse, and obesity has been reported, alongside increased rates of certain conditions, including HIV among some groups of older gay men [16]. Delayed healthcare seeking may further contribute to late diagnosis and poorer outcomes [17].

Mental health has been a central focus in previous research [12,14,15,16], often linked to social relationships and experiences of loneliness, which may lead to depression. Fear of stigma may further exacerbate these challenges [18]. In response, many individuals develop alternative support networks, often referred to as “chosen families,” consisting of friends, partners, and community members. These networks play a crucial role in reducing isolation; however, they may diminish over time due to ageing and loss [19].

Older adults with an LGBT+ identity are also less likely to have established conventional family structures. For some, disclosure of sexual orientation or gender identity may strain relationships with children and other family members, sometimes resulting in estrangement [6,20]. Conversely, having a partner in later life has been associated with improved quality of life and better mental health outcomes [15].

Danish studies show that approximately 28% of LGBT+ individuals experience loneliness, rising to 46% among individuals with a trans identity [21,22,23]. Similarly, suicidal ideation is reported among 44% of lesbian women and 43% of gay men, with even higher rates among bisexual individuals [23]. These figures are significantly higher than among heterosexual populations. However, existing studies rarely include individuals aged 65 years and older. Importantly, older adults with an LGBT+ identity do not constitute a homogeneous group. From an intersectional perspective [24], health outcomes are shaped by the interplay of multiple social positions, including age, sexual orientation, gender identity, socioeconomic status, and cultural or religious context [25,26]. These intersecting factors influence both exposure to minority stress [12] and access to healthcare and social resources, resulting in diverse health trajectories within this population [26,27,28].

A recent Danish nationwide study found that 21.7% of the population expressed moral disapproval of same-sex relationships, particularly among older individuals and those with strong religious beliefs [29]. This suggests that older adults with an LGBT+ identity may still encounter negative attitudes, especially in contexts such as long-term care where dependence on others increases.

Definitions of older age vary globally. The World Health Organization (WHO) defines “older adults” as individuals aged 60–74 years and “elderly” as those aged 75 years and above [30]. However, there is no universally agreed-upon age threshold in academic literature, with some studies including individuals from the age of 50. Such lower thresholds often reflect context-specific considerations, including earlier onset of age-related vulnerabilities in certain populations. In Denmark, approximately 1.2 million individuals are aged 65 years or older, within a total population of 6 million [31], a number projected to increase to 1.4 million by 2030 [32]. The age threshold of 65+ is widely used to define older adults in Denmark [33,34], reflecting its historical link to the statutory retirement age and its continued relevance in policy, health service organization, and population statistics. Although increasing life expectancy is gradually shifting retirement age, 65 years remains a key institutional and analytical threshold [35].

This integrative review aims to synthesize existing research on how older adults 65+ with an LGBT+ identity experience their encounters with the healthcare system. The review focuses on identifying barriers, challenges, and variations in these experiences, as well as highlighting gaps in the current literature to inform future research and practice.

We worked with the following research question:

*How do adults 65 years and older, with an LGBT+ identity experience their encounters with the healthcare system?*

## 2. Methods

### 2.1. Design

An integrative literature search of multiple databases was performed, and studies were screened using predefined inclusion and exclusion criteria. In line with the aim of exploring experiences, only studies containing qualitative data, either as primary qualitative studies or qualitative components within mixed-methods designs were included. Data were analysed using systematic text condensation. The review follows the PRISMA outline [36], which can be seen in the PRISMA checklist (Tables S1 and S2) with a literature search and analysis inspired by Malterud’s text condensation [37]. Included articles focused on the encounter between healthcare professionals and older adults 65+ with an LGBT+ identity, from the perspective of the older adults (Table S3). The project has been registered at the Open Science Framework, on the 13 April 2026, and is, at the time of submission of the article, pending embargo approval.

### 2.2. Literature Search

In this review, we apply an age threshold of 65+ to ensure conceptual clarity and contextual relevance. This cutoff aligns with Danish demographic classifications and welfare structures, thereby strengthening the applicability and comparability of the findings within a Danish context.

Using the principles outlined in PRISMA 2020 [36], an integrative literature search was conducted in the following databases: CINAHL, EMBASE, PubMed, MEDLINE, PsycINFO, and Scopus. The search strategy was developed in collaboration with a research librarian and structured around four conceptual blocks: (1) LGBT+ identity, (2) older age, (3) experiences and perceptions, and (4) healthcare and care contexts. Within each block, a combination of controlled vocabulary (e.g., MeSH terms in PubMed) and free-text terms were applied, and terms were combined using Boolean operators (AND/OR).

The search strategy was designed to capture studies across a range of healthcare settings, including primary care, community-based services, and institutional care. The healthcare-related search terms included both general and primary care contexts, and hospital-based studies. Search strategies were adapted to each database to account for differences in indexing systems and search functionalities. The full search strategies for all databases are provided in Appendix A.

**Figure 1:**
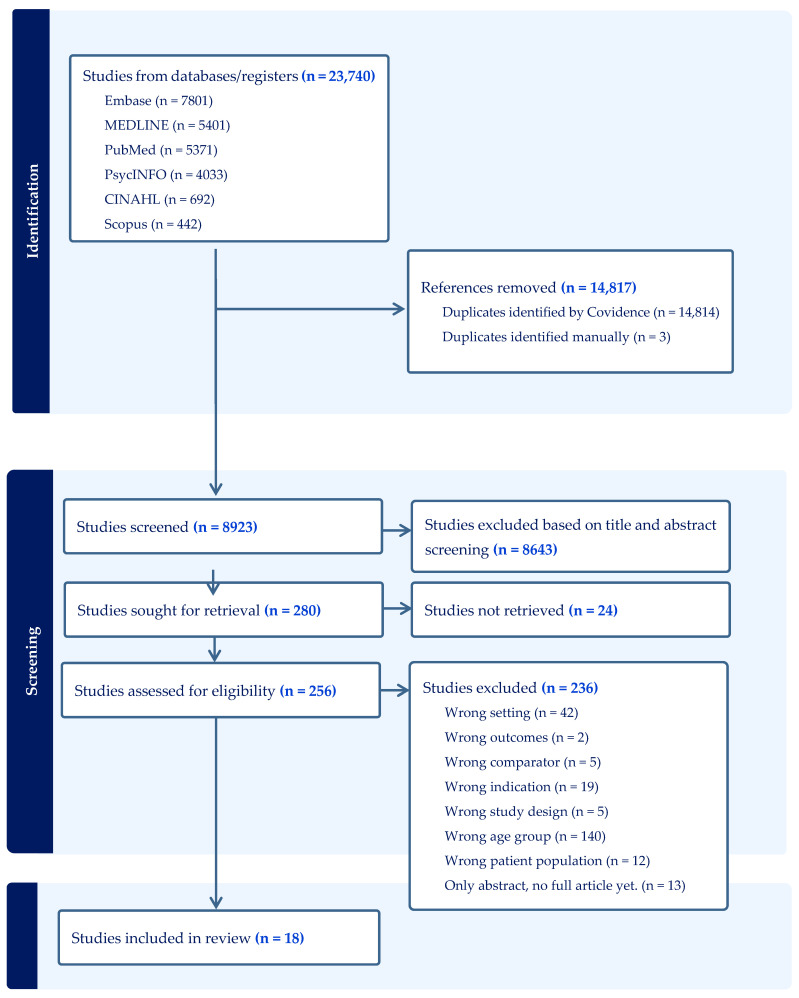
Shows the PRISMA diagram as generated in COVIDENCE.

Searches were conducted initially in May 2021, with updates performed through March 2026 to capture new publications. The PRISMA flow diagram illustrating the study selection process is presented in Figure 1. Eligible articles included those published in English, Danish, Swedish, or Norwegian and available in full-text format. The first author (AVJ) carried out the search under the supervision of a research librarian who also assisted in developing the search strategy.

### 2.3. Inclusion and Exclusion Criteria

Studies were included if they were published after 2010, involved participants aged 65 years or older, and were peer-reviewed to ensure a minimum level of methodological quality. Only studies focusing on the perspectives of older LGBT+ individuals were considered, to maintain a clear focus on the target population. Additionally, eligible studies were required to address interactions with healthcare professionals or healthcare systems. Articles written in English, Danish, Swedish, or Norwegian were included.

Studies were excluded if they focused on perspectives of health professionals or relatives rather than older LGBT+ individuals. Studies involving participants younger than 65 years were also excluded, as the review specifically targeted older adults, defined in this context as individuals aged 65 years and above. Furthermore, educational or teaching papers were excluded, as they did not align with the aim of examining empirical research on lived experiences.

### 2.4. Data Extraction Process and Quality Assessment

Article selection was initially conducted by the first author AVJ and SV through screening of titles and abstracts. Full-text articles were subsequently reviewed by AVJ and SV, applying predefined inclusion and exclusion criteria. The final selection was made jointly by the two authors, and any disagreements were resolved through discussion with a third reviewer (DSN).

The systematic review process was managed using the online software Covidence (https://www.covidence.org/), which facilitated study selection, data extraction, and quality assessment. Following each database search, references were imported into Covidence, where duplicate records were automatically identified and removed. Reviewers then logged in with their individual ID-login, thereby securing anonymity of the reviewing process, and undue influence between reviewers.

Data extraction was performed using Covidence in combination with a standardized data extraction template. The methodological quality of all included studies was assessed using the CASP checklist [38] (Table S4).

Given that the included studies were primarily qualitative in nature, the quality appraisal focused on aspects such as credibility, transferability, and transparency in reporting. Findings from studies assessed as having lower methodological quality were interpreted with caution during the synthesis.

One integrative study was included; however, only findings compatible with qualitative analysis were extracted, ensuring consistency in the analytical approach. No studies were excluded based on quality assessment, but the appraisal informed the interpretation of findings.

The use of Covidence supported a structured, transparent, and reproducible review process.

### 2.5. Analysis

Inspired by Malterud’s systematic text condensation [37], a four-step thematic analysis was conducted in accordance with PRISMA guidelines [36] for systematic synthesis. Although systematic text condensation was originally developed for the analysis of primary qualitative data, it was applied as an analytical strategy for synthesizing textual findings across studies with diverse designs. The analysis focused on the reported experiences and interpretations presented in the included articles, including qualitative findings as well as narrative components from mixed-methods and reviews, rather than on quantitative outcomes. Thus, the unit of analysis consisted of meaning units extracted from results and discussion sections across studies.

In step one, the authors read all included papers to establish an overall impression and gain familiarity with the material, noting preliminary themes and patterns. In step two, a methodical review of the texts was carried out, with meaning units relevant to the research question identified, coded, and categorized, paralleling the PRISMA stages of screening and eligibility assessment. Step three involved condensation, in which the meaning units were abstracted, reduced, and sorted into code groups, allowing for comparison across studies while retaining the context of each original paper. In step four, the condensates were synthesized into coherent analytic text, where categories were developed into concepts and descriptive themes that directly addressed the research question.

In applying this approach, published findings were treated as the data material, enabling a systematic and transparent cross-case analysis while maintaining closeness to participants’ reported experiences. Throughout the process, each paper was analysed systematically and iteratively by the authors, and interpretations were continuously compared and discussed to ensure transparency, consistency, and rigor in the analytical process. We acknowledge that applying systematic text condensation to secondary data differs from its original use in primary qualitative research and involves an additional level of interpretation.

## 3. Results

The total search retrieved 23,740 studies from all databases, with 18 studies included in the final analysis after screening. The screening process is shown in a PRISMA flow diagram (Figure 1).

We analysed the findings through the lens of minority stress theory [12], which posits that health disparities among minority populations arise from chronic exposure to stigma-related stressors. Across the included studies, older persons with an LGBT+ identity described experiences corresponding to both distal stressors, such as discrimination and heteronormative assumptions in healthcare, and proximal stressors, including fear of discrimination, ageism, and concealment of identity. These stressors appear to accumulate across the life course, contributing to healthcare avoidance, delayed diagnosis, and adverse mental health outcomes. At the same time, social networks and chosen families function as important protective factors, although these are often fragile in later life.

An intersectional perspective [24] further deepens the analysis by highlighting how multiple social positions—such as age, sexual orientation, gender identity, and cultural context—interact to shape healthcare experiences. For example, older transgender adults appeared particularly vulnerable, reflecting the compounded effects of ageism and transphobia. Similarly, experiences of exclusion within both healthcare systems and LGBT+ communities point to the complex ways in which marginalisation operates across contexts.

Together, these perspectives support an understanding of healthcare encounters as shaped by the interaction of individual experiences, relational dynamics, and structural conditions, and provide a conceptual framework for interpreting variation and heterogeneity across the findings. In addition, a life-course perspective is reflected in the findings, whereby earlier experiences of stigma, including those related to the AIDS epidemic, continue to influence trust in healthcare systems and expectations of care in later life [39].

From the included articles shown in Table S3, the analysis revealed three overall themes: (1) double-edged discrimination—experiences of stigma and anticipated fear in healthcare; (2) relational networks as essential yet fragile sources of support in later life; (3) healthcare practices as shaping inclusion or invisibility—the need for competence and recognition. In total around 450 participants with an LGBT+ identity were included in the 18 articles.

### 3.1. Theme 1—Double-Edged Discrimination: Experiences of Stigma and Anticipated Fear in Healthcare *[40,41,42,43,44,45,46,47,48,49,50,51,52,53,54,55,56,57]*

The first identified theme concerns older adults with an LGBT+ identity’s fear of and lack of trust in healthcare professionals and systems, shaped by both experiences of discrimination and expectations of future negative encounters [40,41,42,43,44,45,46,47,48,49,50,51,52,53,54,55,56,57].

Participants in the included studies described how they were met with heteronormative language and assumptions in clinical practice [40,41,50,53,55], as well as direct discrimination related to their sexual orientation or gender identity [43,44,47,49]. These experiences reflect how stigma can be embedded in everyday healthcare interactions, influencing how individuals are seen, addressed, and treated. In addition, some participants described experiences of ageism within healthcare, where advanced age led to assumptions of reduced cognitive or physical capacity and resulted in patronizing communication [47,49]. This suggests that discrimination may operate across multiple, intersecting dimensions, including both age and LGBT+ identity.

Across the included studies, fear of discrimination when accessing healthcare services was prominent, particularly among older transgender adults, who were described as experiencing higher levels of fear compared to homosexual individuals [40,41,42,43,44,45,46,49,50,52,53,54,55,57]. In some studies, this fear was associated with increased stress and poorer mental health outcomes [42,46,54], indicating that not only experienced but also anticipated discrimination may influence well-being. The findings also reveal important intra-group variations in experiences of discrimination and fear. For example, one study highlighted how trans men reported heightened fear of discrimination and stigma, particularly in relation to sexual and reproductive healthcare services, which were often perceived as female-centred and normatively gendered. In contrast, trans women in the same study described more frequent use of self-advocacy strategies when engaging with healthcare professionals, suggesting differing ways of navigating potentially exclusionary environments [53]. These differences illustrate how experiences of healthcare are not only shaped by LGBT+ identity broadly but also by specific gender identities, care contexts, and expectations embedded within healthcare structures.

Several studies also highlighted how past experiences continue to shape present perceptions of healthcare [40,41,42,46,49,52,53]. Many older adults with an LGBT+ identity had lived through the AIDS epidemic and described enduring memories of stigma, taboo, and exclusion within healthcare systems [56]. These life-course experiences appeared to contribute to ongoing mistrust and expectations of discrimination.

Fear of discrimination was further described in relation to future care needs. End-of-life planning, potential relocation to retirement homes, or increasing dependence on care services often evoked concerns about having to conceal one’s identity—referred to as “going back into the closet” [40,44,45,48,50,51,52,56,57]. As a result, some participants emphasized the importance of accessing LGBT+-friendly services [40,42,43,44,45,46,47,48,53,56] or relying on chosen family and support networks when engaging with healthcare [43,45,50]. Importantly, the degree and nature of fear were not uniform across participants. Several studies highlighted that older transgender adults experienced more pronounced concerns related to discrimination compared to gay and lesbian individuals, suggesting that vulnerability may vary across subgroups within the LGBT+ population [40,41,43,44,45,46,49,50,52,53,55,57]. These findings reflect both proximal and distal stressors, where not only direct experiences of discrimination but also anticipated stigma shape healthcare engagement.

The studies suggest that both experiences of discrimination and fear of future discrimination may lead to reluctance in seeking healthcare or disclosing one’s LGBT+ identity [50,52,53,56]. This may contribute to delayed diagnosis and disparities in health outcomes, including mental health challenges, higher substance use, and increased prevalence of certain conditions among some groups within the LGBT+ population [55]. These patterns align with the notion that cumulative exposure to stigma can have long-term consequences for health. While discrimination was a recurring finding, it took various forms dependent on context, ranging from subtle heteronormative assumptions in everyday interactions to more explicit instances of prejudice [41,47,52]. This variation indicates that exclusion in healthcare may operate both overtly and more implicitly through normative practice.

Finally, several studies highlighted concerns related to loss of autonomy, identity, and the ability to self-advocate, particularly in later life and in more dependent care situations [40,41,48,50,52,57]. These concerns further reinforce the importance of understanding how fear and previous experiences shape healthcare engagement among older adults with an LGBT+ identity.

Taken together, the findings illustrate how both experienced and anticipated discrimination contribute to shaping healthcare engagement among older adults with an LGBT+ identity. While fear and mistrust were common across studies, important variations were evident across subgroups and contexts, particularly in relation to gender identity and care settings. This highlights that experiences of discrimination are not uniform but are shaped by intersecting factors and prior life experiences.

### 3.2. Theme 2—Relational Networks as Essential Yet Fragile Sources of Support in Later Life *[40,42,43,44,45,46,47,48,49,50,51,52,54,55,57]*

The second identified theme concerns the importance of relational and social networks for older adults with an LGBT+ identity, particularly as sources of emotional, social, and practical support in relation to healthcare [40,42,43,44,45,46,47,48,49,50,51,52,54,55,57].

Across the included studies, many older adults with an LGBT+ identity were described as having had limited opportunities earlier in life to establish what are often considered conventional family structures, such as long-term partnerships or parenthood, due to legal and societal barriers [49,52]. Instead, many had formed so-called “chosen families”, consisting of friends, partners, both current and former and colleagues [42,43,44,45,46,48,50,51,52,55]. These networks were frequently described as central sources of support in later life.

However, the findings indicate important differences in the composition and function of social networks across subgroups. Among transgender participants, networks were often described as smaller or, in some cases, absent, which may increase vulnerability in relation to healthcare access and support [40,52,54]. In contrast, studies including broader LGBT+ populations more frequently described larger and more diverse networks, encompassing both chosen families and, in some cases, biological family connections. This pattern is not uniform. Some studies highlighted difficulties in establishing new social connections in later life, partly due to experiences of ageism within LGBT+ communities themselves [48,51]. These differences were also reflected in how support was mobilised. Transgender participants appeared to rely more heavily on trans-specific support groups, particularly when seeking appropriate healthcare and navigating gender-affirming services [53]. By contrast, studies focusing on broader LGBT+ populations more often described reliance on informal caregivers within existing networks, including partners, friends, and chosen family members [43,44,47].

At the same time, several studies highlighted that relationships with biological families were often limited or absent, primarily due to lack of acceptance of the individual’s sexual identity [40,42,43,46,48,49,52]. For those who had children, relationships were sometimes described as fragile, with some older adults feeling compelled to conceal their identity to maintain contact [40,43,48,55]. These findings point to a particular form of vulnerability, where reduced access to traditional family support may increase reliance on alternative networks.

Chosen families and broader social networks were described as playing a crucial role in providing informal care, emotional support, and protection against loneliness and social isolation [42,43,47,50,52,55]. Several studies linked the presence—or absence—of such networks to mental health outcomes, suggesting that limited social support may contribute to increased loneliness and decreased well-being [43,45,46,48,49,51]. In this way, relational networks appear to function as important resources that may help mitigate some of the challenges associated with ageing and healthcare access. However, the reliance on chosen families also reflects structural conditions, where limited access to traditional family support shifts the responsibility for care and support onto alternative networks.

In the context of healthcare, networks were also described as important for safeguarding autonomy and identity, particularly in situations involving hospitalization or long-term care. Several studies emphasized the role of advance care planning, including establishing living wills, appointing healthcare proxies, and identifying individuals allowed to visit, as ways of ensuring that chosen family members are recognized and included in care decisions [42,50,52,57]. These strategies were described as particularly important given concerns about discrimination and exclusion within healthcare settings.

However, the findings also illustrate that these networks may be fragile and subject to change over time. Many older adults with an LGBT+ identity had experienced the loss of significant members of their social circles, particularly during the AIDS epidemic, which had a lasting impact on the size and stability of their networks [54,56]. In addition, in several studies participants described difficulties in establishing new relationships within the LGBT+ community in later life, partly due to experiences of ageism within the community itself [46,48,49,51,54]. The stability and availability of these networks were not consistent across groups. Several studies indicated that bisexual and transgender individuals often had smaller or less stable networks compared to lesbian and gay individuals, suggesting unequal access to social support within the LGBT+ population [40,45,48,51]. These findings point to the long-term impact of life-course experiences, where earlier losses and social disruptions continue to shape the availability of support in later life.

Overall, this theme highlights how relational networks constitute essential yet potentially vulnerable sources of support in later life. While chosen families and social connections can provide care, protection, and a sense of belonging, their fragility and variability may also contribute to increased vulnerability, particularly in the absence of formal recognition within healthcare systems. Relational networks emerged as both essential and vulnerable sources of support. While chosen families and social connections may mitigate isolation and support healthcare navigation, their fragility and unequal distribution across subgroups may also contribute to increased vulnerability. This underscores the importance of understanding social support not only as a resource but also as a factor shaped by broader social and historical conditions. Together, these findings suggest that both the availability and type of social support vary across subgroups, influencing how older persons with an LGBT+ identity access care, seek support, and manage healthcare encounters.

From a minority stress perspective, these networks may function as protective resources, although their variability and fragility suggest unequal access to such buffering mechanisms across subgroups.

### 3.3. Theme 3—Healthcare Practices as Shaping Inclusion or Invisibility: The Need for Competence and Recognition *[41,43,44,45,46,48,50,51,52,53,54]*

The third identified theme concerns the role of healthcare professionals and care practices in shaping the experiences of older adults with an LGBT+ identity, particularly in relation to recognition, inclusion, and quality of care [41,43,44,45,46,48,50,51,52,53,54].

When seeking healthcare services, many older adults with an LGBT+ identity reported a preference for LGBT+-inclusive services, particularly in long-term care settings [41,43,44,45,46,48,50,51,52,53,54]. One study mentioned that if staff had gay or lesbian acquaintances, they would be less likely to be homophobic [45].

Two papers illustrated that healthcare professionals frequently assert that they “treat everyone equally” [41,45], implying that care was delivered without consideration of patients’ sexual orientation or identity. Nevertheless, this approach may render LGBT+ identities invisible within healthcare settings and contribute to the systematic neglect of health issues uniquely affecting older adults with an LGBT+ identity. Consequently, both the specific healthcare needs of this population and significance of their support networks and partners risked being overlooked, potentially limiting the effectiveness and inclusivity of care [41,43,45].

The findings also indicate important differences in how openness and disclosure influence healthcare experiences across subgroups. Several studies focusing on lesbian and broader LGBT+ populations suggest that openness about sexual orientation does not necessarily affect the level of care received [43,44,47].

In contrast, studies focusing on transgender participants reveal a different dynamic. For many transgender individuals, disclosure is often unavoidable, particularly in clinical contexts related to gender identity or medical history [40,49,50,52,53,54]. In these cases, openness is not a choice but a structural condition of care, which may increase exposure to discrimination. Indeed, transgender participants reported more frequent experiences of stigma and unequal treatment, even when engaging openly with healthcare professionals [40,49,50,52,53,54].

This contrast highlights a key tension within healthcare encounters: while openness may be perceived as neutral or even beneficial in some contexts, it may simultaneously increase vulnerability in others. Studies focusing on older transgender adults emphasise the importance of transgender-affirming care, suggesting that such approaches are essential for maintaining trust and ensuring continued engagement with healthcare services [52,53].

These dynamics illustrate how healthcare practices may either reinforce or mitigate structural forms of stigma, depending on how identities are recognised and accommodated in care settings.

Another recurrent theme was the need for culturally competent healthcare and enhanced training for healthcare professionals [46,50,51,53,54]. Some participants suggested that healthcare providers, particularly within long term care, lack adequate preparation to address the needs of older adults with an LGBT+ identity. Proposed strategies to improve training included education on sexuality, gender, discrimination, and LGBT+ relationships, as well as the development of communication skills—particularly the use of inclusive language—and initiatives aimed at fostering LGBT+-affirming environments within long-term care settings [40,44,45].

Only one paper focused on religious backgrounds and illustrated that this also could have negative impact on the level of care provided [44]. The primary consequence of homecare workers having a different religious background, or being overly religious, was a negative influence on the older adults with an LGBT+ identity and their relationship with the homecare worker, as well as experiencing instances of discrimination and homophobia from the homecare worker. This often led to either the participant ending the relationship, or the homecare worker quitting, or the participant hiding their identity. One participant described “de-gaying” her home due to her African homecare worker, and a presumption that due to the difference in culture, her homecare worker would not be accepting of LGBT+ persons [44].

This theme highlights how healthcare practices and professional competencies play a central role in either reinforcing or mitigating experiences of exclusion and uncertainty. When healthcare professionals lack awareness or rely on assumptions of sameness, this may contribute to invisibility and unmet needs. Conversely, culturally competent and inclusive practices may support recognition, trust, and more equitable care for older adults with an LGBT+ identity.

These findings illustrate how the implications of openness are context-dependent and shaped by both identity and healthcare structures, underscoring the need for differentiated and inclusive approaches to care.

### 3.4. To Summarize

Healthcare practices play a central role in shaping whether care environments are experienced as inclusive or exclusionary. While some approaches aim to ensure equal treatment, they may inadvertently contribute to the invisibility of LGBT+ identities and unmet needs. At the same time, variations in knowledge, attitudes, and cultural contexts suggest that healthcare encounters are shaped by both individual experiences and structural conditions.

Across the three themes, the findings reveal both shared patterns and important differences in how older persons with an LGBT+ identity experience and navigate healthcare. Experiences of discrimination, access to social support, and interactions with healthcare professionals were closely interconnected, while also varying across subgroups, care contexts, and life-course experiences.

Together, these findings illustrate how healthcare experiences are shaped by a dynamic interplay of minority stress processes, relational resources, and structural factors, supporting a more integrated and theoretically informed understanding.

## 4. Discussion

This review synthesized evidence on how older adults with an LGBT+ identity experience encounters with healthcare. Across the three themes, the findings demonstrate that healthcare engagement is shaped by a complex interplay of discrimination, social support, and healthcare practices. While these patterns were consistent across studies, important variations emerged across subgroups, care contexts, and life-course experiences.

To further interpret these findings, the results can be understood through the lens of minority stress theory, which explains how health disparities among sexual and gender minority populations arise from chronic exposure to stigma, discrimination, and social exclusion [44,58,59,60]. Recent developments in minority stress research emphasize not only the persistence of these stressors but also the mechanisms through which they influence mental and physical health, including affective, cognitive, and social processes [14,16,28,61]. The experiences identified in this review, such as fear of discrimination, concealment of identity, and delayed healthcare seeking, reflect both external stressors (e.g., discriminatory healthcare encounters) and internal or anticipated stress processes. These stressors have been consistently linked to poorer mental health outcomes and increased health risks among LGBT+ populations [16,62,63,64,65,66].

In addition, an intersectional perspective provides further insight into the variation observed across subgroups within the LGBT+ population [63,64,67,68]. Recent research highlights how multiple and intersecting forms of marginalisation including gender identity, age, and social context, shape exposure to minority stress and access to healthcare resources [24,67,68,69,70,71]. The findings of this review suggest that transgender and gender-nonconforming older adults may experience particularly intensified barriers, reflecting how intersecting vulnerabilities influence healthcare access and outcomes [40,41,50,52,53,54]. Together, these perspectives support an understanding of healthcare experiences as shaped by a dynamic interplay of structural, relational, and individual factors, rather than as uniform across the population.

The findings also point to important differences between types of social networks. Biological families were often described as fragile or absent due to experiences of non-acceptance, while chosen families—including friends, partners, and community members—played a central role in providing emotional and practical support [40,42,44,48,49,51,55]. In addition, community-based networks were important for maintaining identity and belonging, although access to such networks varied [47,52,54].

These different forms of support also serve distinct functions in healthcare contexts. Social networks may act as sources of trust and emotional security, facilitate advocacy during healthcare encounters, and support navigation of complex healthcare systems [54,72,73,74]. At the same time, reliance on chosen and community-based networks may introduce vulnerabilities, as these networks are often informal, may diminish over time, and are not always recognized within healthcare structures [75,76].

Importantly, the findings demonstrate that older adults with an LGBT+ identity are not a homogeneous group. Subgroup analyses revealed that transgender and gender-nonconforming individuals often face more pronounced barriers, particularly in relation to discrimination, access to care, and smaller or less stable social networks [40,41,50,52,53,54]. Similarly, bisexual individuals were described as having less access to support networks compared to lesbian and gay individuals [40,45,47,48,51]. These differences highlight the need to move beyond a unified understanding of “older LGBT+ adults” and instead adopt a more nuanced and intersectional perspective.

Context also played a significant role. The majority of the included studies focused on long-term care settings [40,43,44,45,47,51], while fewer examined primary care or hospital-based encounters [40,41,46,50,52,53]. This imbalance suggests that current knowledge is shaped by a particular institutional context, potentially overlooking important dynamics in other parts of the healthcare system. Experiences of vulnerability and fear appeared to intensify in settings where individuals anticipated loss of autonomy, such as long-term care, whereas less is known about how these dynamics unfold in more episodic or acute care contexts.

At the same time, the findings reveal important tensions within healthcare practices. For example, the principle of “treating everyone equally” was described as both supportive and problematic [9,43,77]. While intended to promote fairness, it may contribute to the invisibility of LGBT+ identities and specific care needs. This highlights a broader structural challenge in healthcare systems, where equality-based approaches may fail to account for differences and thereby reproduce inequities [26,78].

To further deepen the interpretation of the findings, the results can be understood across micro-, meso-, and macro-level dimensions. At the micro level, the findings highlight how individual experiences of fear, concealment, and anticipated discrimination shape healthcare engagement [40,46,49,55]. These processes reflect both internalised and anticipated aspects of minority stress, which have been shown to influence mental and physical health outcomes among sexual and gender minority populations [79]. For many older adults with an LGBT+ identity, previous experiences of stigma contribute to ongoing vigilance and reluctance to engage fully with healthcare services.

At the meso level, the findings point to the importance of interpersonal relationships and organisational practices within healthcare settings [41,42,43,44,45,47,51,52,53,54,57]. Interactions with healthcare professionals, including communication styles, recognition of chosen families, and professional competence, play a critical role in shaping whether care is experienced as safe and inclusive. Recent research highlights that lack of provider knowledge, implicit bias, and insufficient training remain key barriers to equitable care for LGBT+ older adults [80,81]. These findings underscore how organisational cultures and everyday practices within healthcare institutions can either mitigate or reinforce minority stress.

At the macro level, the findings reflect broader structural and societal influences, including policy frameworks, cultural norms, and historical experiences of marginalisation [26,43,44,45,47,48,50,51,52,55,56]. Intersectional research increasingly demonstrates how multiple forms of marginalisation, such as age, gender identity, and ethnicity—interact to produce unequal health outcomes and access to care [79,82]. In addition, population-based studies suggest that while disparities exist, they are not uniform, as resilience and social determinants of health may buffer or amplify the effects of minority stress [27,83]. The predominance of North American studies in this review further reflects how findings are embedded within specific policy and healthcare systems, highlighting the need for broader international research.

Taken together, this multi-level perspective illustrates how healthcare experiences among older adults with an LGBT+ identity are shaped by the interaction of individual, relational, and structural factors. Addressing inequalities in healthcare therefore requires coordinated interventions across all three levels, including individual support, organisational change, and policy development.

## 5. Limitations

This review has several limitations that should be considered when interpreting the findings.

First, the included studies were predominantly conducted in North America [40,42,43,44,45,46,48,49,50,52,53,54,55,56,57], particularly the United States [40,42,43,44,45,46,50,52,53,54,55,56,57]. This geographical concentration may limit the transferability of findings, as healthcare experiences are shaped by national policies, welfare systems, and cultural norms. For example, healthcare systems in Scandinavian countries are characterised by universal access and different approaches to social care, which may influence both experiences of discrimination and expectations of care [3,83,84]. Consequently, the findings may reflect context-specific dynamics rather than universally applicable patterns, highlighting the need for more geographically diverse research.

Second, the restriction to studies including participants aged 65 years and older, while contextually grounded in Danish definitions of older age [34], may have influenced the scope of the included literature. Many international studies apply a 50+ threshold [25], often reflecting earlier exposure to health inequalities among LGBT+ populations. As a result, relevant insights from younger cohorts—who may already experience ageing-related vulnerabilities, may have been excluded. This choice may therefore contribute to a narrower understanding of ageing within LGBT+ populations and should be considered when interpreting the findings.

Third, although only studies containing qualitative data were included to ensure alignment with the aim of exploring experiences, variation in methodological quality and design remained. Differences in sampling strategies, analytical approaches, and reporting transparency may influence how experiences are represented across studies. While quality appraisal informed the interpretation of findings, this heterogeneity may still affect the depth and comparability of the synthesis.

Fourth, the predominance of cross-sectional study designs limits insight into how healthcare experiences evolve over time. As a result, the findings primarily reflect experiences at specific points in time and may not fully capture changes related to ageing, health status, or shifting social contexts.

Finally, publication bias cannot be excluded. Studies reporting experiences of discrimination, marginalisation, or unmet needs may be more likely to be published, potentially leading to an overrepresentation of negative healthcare experiences. In addition, the exclusion of grey literature and studies published in other languages may have limited the inclusion of alternative perspectives, particularly from non-Western contexts.

## 6. Conclusions

This integrative review demonstrates that healthcare encounters among older persons with an LGBT+ identity are shaped by a complex interplay of fear, prior experiences of discrimination, and expectations of stigma, which together influence healthcare engagement, disclosure, and timing of care. While these barriers are well documented, this review highlights how they are embedded in life-course experiences and continue to affect interactions with healthcare systems in later life.

Importantly, the findings reveal that healthcare experiences are not uniform but vary across subgroups and contexts. Particularly transgender and bisexual older adults appear to face more pronounced barriers, while differences in access to and reliance on social networks further shape healthcare navigation and support. Social networks—especially chosen families—play a crucial role in mediating trust, advocacy, and access to care, yet they also represent potentially fragile and informal structures that are not consistently recognised within healthcare systems.

The review further identifies key tensions within healthcare practices, including how approaches based on “equal treatment” may inadvertently contribute to the invisibility of LGBT+ identities and specific care needs. This highlights the importance of moving beyond generalised approaches toward more inclusive and context-sensitive care.

By applying a qualitative and interpretive synthesis supported by theoretical perspectives, this review contributes to a more nuanced understanding of how individual, relational, and structural factors interact to shape healthcare experiences. The findings underscore the need for coordinated efforts across levels, including targeted training, recognition of diverse support networks, and structural changes within healthcare systems.

## 7. Policy and Practice Implications

The findings have important implications for healthcare practice and policy. First, there is a clear need for systematic and mandatory training of healthcare professionals across all levels of care. Such training should include knowledge of LGBT+ health needs, inclusive communication, and awareness of life-course experiences. To enhance effectiveness, training may incorporate case-based learning, reflective practices, and co-production with older persons with an LGBT+ identity.

Second, healthcare systems should implement structural measures to support inclusivity, including the use of inclusive language, recognition of chosen families, and the development of LGBT+-affirming care environments—particularly in long-term care settings. These initiatives should be embedded within organisational policies and professional education.

Third, policy initiatives should address institutional barriers, ensuring that care standards and guidelines explicitly reflect the needs of older adults with an LGBT+ identity. The effectiveness of such interventions should be evaluated using measurable outcomes, including changes in healthcare professionals’ competencies and patient-reported experiences of trust, safety, and access to care.

Finally, the findings highlight that a “one-size-fits-all” approach is insufficient. Targeted strategies are needed for subgroups, particularly transgender and bisexual older adults, who may face distinct barriers and require tailored support.

## 8. Recommendations for Future Research

Future research should prioritise:

- Greater inclusion of diverse subgroups, particularly transgender, bisexual, and ethnic minority older adults
- Studies across a broader range of healthcare settings, including primary and hospital care
- Longitudinal research to better understand how healthcare experiences evolve over time.
- Intervention studies evaluating the effectiveness of training and inclusive care models

In addition, there is a need for research that explicitly examines structural and policy-level factors influencing healthcare access and experiences.

## Data Availability

No new data were created or analysed in this study. Data sharing is not applicable to this article.

## Supplementary Materials

The following supporting information can be downloaded at: https://www.mdpi.com/article/10.3390/healthcare14081110/s1, Table S1: PRISMA abstract checklist, Table S2: PRISMA checklist, Table S3: Included articles; Table S4: CASP-checklist. Ref. [85] was cited in Supplementary Materials.

## Appendix A

### Appendix A.1. Search Strategy

**Table A1:** PRISMA Search table.

Search Engine	Search Strategy
PubMed	(“Sexual and Gender Minorities”[Mesh] OR LGBT*[tiab] OR homosexual*[tiab] OR gay[tiab] OR lesbian*[tiab] OR bisexual*[tiab] OR transgender*[tiab] OR transsexual*[tiab] OR queer*[tiab] OR “sexual minorit*”[tiab] OR “gender minorit*”[tiab] OR nonbinary[tiab] OR “non-binary”[tiab]) AND (“Aged”[Mesh] OR “Aged, 80 and over”[Mesh] OR elderly[tiab] OR older[tiab] OR aging[tiab] OR ageing[tiab] OR “older adult*”[tiab] OR senior*[tiab] OR geriatric*[tiab] OR “later life”[tiab]) AND (“Perception”[Mesh] OR “Social Interaction”[Mesh] OR “Interpersonal Relaions”[Mesh] OR experience*[tiab] OR perception*[tiab] OR perspective*[tiab] OR encounter*[tiab] OR “lived experience*”[tiab]) AND (“Health Services”[Mesh] OR “Delivery of Health Care”[Mesh] OR “Health Facilities”[Mesh] OR “Hospitals”[Mesh] OR “Primary Health Care”[Mesh] OR “Ambulatory Care”[Mesh] OR hospital*[tiab] OR “primary care”[tiab] OR healthcare[tiab] OR “health care”[tiab])
Medline (Ovid)	1. exp Sexual and Gender Minorities/2. (LGBT* OR homosexual* OR gay OR lesbian* OR bisexual* OR transgender* OR queer* OR “sexual minorit*” OR “gender minorit*” OR nonbinary OR “non-binary”).ti,ab.3. 1 OR 2 4. exp Aged/5. (elderly OR “older adult*” OR aging OR ageing OR senior* OR geriatric* OR “later life”).ti,ab. 6. 4 OR 5 7. exp Perception/ OR exp Interpersonal Relations/ OR exp Social Interaction/8. (experience* OR perception* OR perspective* OR encounter* OR “lived experience*”).ti,ab.9. 7 OR 8 10. exp Health Services/ OR exp Hospitals/ OR exp Primary Health Care/ OR exp Ambulatory Care/ 11. (hospital* OR “primary care” OR healthcare OR “health care”).ti,ab. 12. 10 OR 11 13. 3 AND 6 AND 9 AND 12
PsychINFO (Ovid)	1. exp sexual minorities/ 2. (LGBT* OR homosexual* OR gay OR lesbian* OR bisexual* OR transgender* OR queer* OR “gender minority*” OR nonbinary).ti,ab. 3. 1 OR 2 4. exp aging/ 5. (elderly OR “older adult*” OR senior* OR geriatric* OR “later life”).ti,ab. 6. 4 OR 5 7. exp perception/ OR exp interpersonal relationships/ 8. (experience* OR perception* OR perspective* OR “lived experience*”).ti,ab. 9. 7 OR 8 10. exp health care services/ OR exp hospitals/ 11. (hospital* OR healthcare OR “health care”).ti,ab. 12. 10 OR 11 13. 3 AND 6 AND 9 AND 12
Scopus	TITLE-ABS-KEY(LGBT* OR homosexual* OR gay OR lesbian* OR bisexual* OR transgender* OR queer* OR “sexual minority*” OR “gender minority*” OR nonbinary OR “non-binary”) AND TITLE-ABS-KEY(elderly OR “older adult*” OR aging OR ageing OR senior* OR geriatric* OR “later life”) AND TITLE-ABS-KEY(experience* OR perception* OR perspective* OR encounter* OR “lived experience*” OR interpersonal relation*” OR “social interaction”) AND TITLE-ABS-KEY(hospital* OR primary care” OR healthcare OR “health care”)
CINHAL (EBSCO)	S1 (MH “Sexual and Gender Minorities+”) S2 (LGBT* OR homosexual* OR gay OR lesbian* OR bisexual* OR transgender* OR queer* OR “sexual minorit*” OR “gender minorit*” OR nonbinary OR “non-binary”) S3 S1 OR S2 S4 (MH “Aged+”) S5 (elderly OR “older adult*” OR aging OR ageing OR senior* OR geriatric* OR “later life”) S6 S4 OR S5 S7 (MH “Perception+”) OR (MH “Interpersonal Relations+”) OR (MH “Social Interaction+”) S8 (experience* OR perception* OR perspective* OR encounter* OR “lived experience*”) S9 S7 OR S8 S10 (MH “Health Services+”) OR (MH “Hospitals+”) OR (MH “Primary Health Care+”) S11 (hospital* OR “primary care” OR healthcare OR “health care”) S12 S10 OR S11 S13 S3 AND S6 AND S9 AND S12
Embase (Ovid)	1. exp sexual and gender minority/ 2. (LGBT* OR homosexual* OR gay OR lesbian* OR bisexual* OR transgender* OR queer* OR “sexual minorit*” OR “gender minorit*” OR nonbinary OR “non-binary”).ti,ab. 3. 1 OR 2 4. exp aged/ 5. (elderly OR “older adult*” OR aging OR ageing OR senior* OR geratric* OR “later life”).ti,ab. 6. 4 OR 5 7. exp perception/ OR exp interpersonal communication/ OR exp social interaction/ 8. (experience* OR perception* OR perspective* OR encounter* OR “lived experience*”).ti,ab. 9. 7 OR 8 10. exp health care system/ OR exp hospital/ OR exp primary health care/ 11. (hospital* OR “primary care” OR healthcare OR “health care”).ti,ab. 12. 10 OR 11 13. 3 AND 6 AND 9 AND 12

### Appendix A.2. Search History

**Database**	**Date**	**Search Phase**	**Records Retrieved**	**Records After Deduplication**
PubMed	5 November 2023	Initial search	1540	1512
CINHAL	5 November 2023	Initial search	287	203
MEDLINE	5 November 2023	Initial Search	57	57
PubMed	5 November 2023	Supplementary search	899	860
MEDLINE	7 November 2023	Supplementary search	100	38
Embase	15 March 2024	Expanded search	2838	1415
MEDLINE	15 March 2024	Expanded search	1743	280
PubMed	15 March 2024	Expanded search	273	68
PubMed	14 October 2024	Update search	61	23
MEDLINE	14 October 2024	Update search	401	97
Embase	14 October 2024	Update search	738	152
Scopus	14 October 2024	Update search	57	11
PsycINFO	14 October 2024	Update search	1889	267
PubMed	1 June 2025	Update search	59	54
Embase	1 June 2025	Update search	559	248
MEDLINE	1 June 2025	Update search	439	6
PsycINFO	1 June 2025	Update search	907	84
CINAHL	1 June 2025	Update search	5	1
Scopus	1 June 2025	Update search	13	2
PubMed	26 March 2026	Final update	1221	580
MEDLINE	26 March 2026	Final update	668	4
Embase	26 March 2026	Final update	814	356
CINAHL	26 March 2026	Final update	106	38
Scopus	26 March 2026	Final update	372	90
PsycINFO	26 March 2026	Final update	237	99
