# Supplementary material for: Healthcare Experiences of Older Adults with an LGBT+ Identity: An Integrative Review
Source: Healthcare (Basel). 2026 Apr 21;14(8):1110. doi: 10.3390/healthcare14081110 (PMC13115870; doi:10.3390/healthcare14081110)
Supplement: Supplementary file 1 [file healthcare-14-01110-s001.zip › Table S3- Included studies.pdf]

Title	Lead Authors	Country	Year	Aim	Study Method	Number og Participants	Findings
**Worry and Wisdom: A Qualitative Study of Transgender Elders' Perspectives on Aging**	Adan, Matthew; Scribani, Melissa; Tallman, Nancy; Wolf-Gould, Christopher; Campo-Engelstein, Lisa and Gadomski, Anne	United States	2021	Exploring the perspectives of transgender individuals ages 65 and above on health care, expectations of aging, concerns for the furture, and advice for the next generation.	Semi-structured interviews	19	Seven themes emerged: fear of elder care mistreatment; isolation and loneliness; financial vulnerability; reduced life agency; healthcare discrimination; giving back to the community; and embracing self-truth for personal fulfillment.
**Challenging cisgenderism in the ageing and aged care sector: Meeting the needs of older people of trans and/or non-binary experience**	Ansara, Y. Gavriel	Australia	2015	To explore how applying the cisgenderism framework in ageing and aged care contexts can meet the practical needs of people of trans and/or non-binary experience.	Qualitative data from consultations	Not defined	The study examined the impact of cisgenderism—the ideology invalidating individuals' own understanding of their gender and body—on older LGBT+ care. Participants reported experiences of pathologizing, misgendering, marginalization, coercive queering, and objectifying language, which contributed to distrust in care later in life.
**"Things Are Different Now But": Older LGBT Adults' Experiences and Unmet Needs in Health Care**	Burton, Candace W.; Lee, Jung-Ah; Waalen, Anders and Gibbs, Lisa M.	United States	2020	To explore the local population of older adults' perception of experiences with providers including physicians, nurses	Semi-structured individual interveiws	10	Three themes emerged: Outness, reflecting participants' considerations of their sexuality in relation to health, social life, and disclosure; Social Climate: Things Are Different Now,

				and other caregivers.			highlighting past discriminatory experiences; and Additional Resources, encompassing challenges beyond healthcare, such as securing rights for themselves and partners. Past experiences influenced interactions with healthcare professionals and decisions around disclosure.
**Older lesbians' experiences with home care: Varying levels of disclosure and discrimination**	Butler, Sandra S.	United States	2017	To research the experiences of older lesbians when it comes to long-term services and supports	Semi-structured telephone interviews	31	Four primary themes emerged: Level of disclosure, reflecting LGBT+ individuals' ongoing decisions about whether, when, and how to reveal their identity, with subthemes of "Did not spell out," "No idea I'm lesbian," and "Disclosure up front"; Experiences of homophobia, ranging from tolerance to direct discrimination in home care; Evaluation of care, reporting both satisfaction and dissatisfaction with received services; and Visions of ideal long-term services and supports (LTSS), emphasizing affordable, competent, and inclusive care. The study highlights the need for further research.
**Older lesbians receiving home care:**	Butler, Sandra S.	United States	2018	The article examines experiences of 65	Semi-structured	20 primary 6 partners	Most participants received both formal and informal

**formal and informal dimensions of caregiving**				and older lesbians dealings with formal and informal home care	telephone interviews		care, primarily from partners. Over a third experienced isolation due to limited social networks and prior homophobia. About a quarter reported homophobia from formal healthcare workers, though most eventually developed positive relationships with care providers.
**LGBTQ Older Adults in Long-Term Care Settings: An Integrative Review to Inform Best Practices**	Fasullo, Katherine; McIntosh, Erik; Buchholz, Susan W.; Ruppar, Todd and Ailey, Sarah	United States	2022	To synthesize literature about LGBTQ older adults in long-term care facilites and provide recommedations for best practice guideline development to inform practice, research and policy.	Integrative review including qualitative, quantitative and mixed-method.	20	Older LGBT+ participants highlighted fears of discrimination and “returning to the closet” in long-term care facilities, the importance of social networks, and the need for continuous caregiver education. Caregivers often lacked awareness of LGBT+–specific challenges, treating all residents the same. The study recommends further training, collaboration with LGBT+ organizations, and inclusive policies.
**Meeting the Behavioral Health Needs of LGBT Older Adults**	Goldhammer, Hilary; Krinsky, Lisa and Keuroghlian, Alex S	United States	2019	The aim of the study is to identify barriers that LGBT+ people face, and how clinicians can adress these challenges.	Case report	N/A	The case-study highlighted older LGBT+ individuals’ fears of discrimination in healthcare and workplaces, experiences of living a hidden life, and reliance on chosen families, which may shrink with age. Positive caregiver interactions, including

							respectful presentation and engagement, were shown to benefit the health and well-being of older LGBT+ patients.
**Experiences of older LGBT people ageing in place with care and support: A window on ordinary ageing environments, home-making practices and meeting activities**	Hoekstra-Pijpers, Roos	Netherlands	2022	To research the experiences of older LGBT+ persons who are ageing in place, and who are weekly and monthly recievers of health an social care services.	Survey of 115 participants with 10 follow-up interviews	115	The study identified three overarching themes: (1) aging in place in ordinary ageing environments, (2) aging in place and receiving care at home, and (3) challenges related to the inclusion of older LGBT individuals in LGBT community and neighbourhood-based activities.<br>Most participants reported aging in place, with few considering relocation. Approximately 30% of respondents reported experiences of discrimination from formal caregivers, while slightly more than half received informal care. The majority of respondents were open about their sexual orientation, and various strategies for disclosure to formal caregivers were described.<br>Engagement in LGBT-specific community activities varied, with identified barriers related to inclusion in both

							LGBT and neighbourhood-based contexts.
**"I want to grow older with dignity": Older LGBTQ+ Canadian adults' perceptions and experiences and aging**	Hurd, Laura and; Li, Lynda.Y.K.	Canada	2023	The aim of the study is to examine older LGBTQ+ experiences of aging.	In depth qualitative interviews	30	Three overarching themes emerged regarding losses, gains, and needs among older LGBTQ+ adults. Losses included physical and cognitive health decline, reduced independence, and loneliness or barriers to social engagement, compounded by limited LGBTQ+–inclusive services. Gains encompassed increased wisdom and self-confidence, financial and social flexibility, and support from chosen families and community. Needs highlighted the importance of inclusive, gender-affirming care, meaningful occupation, and strong social connections. The study also emphasized the need for professional training in LGBTQ+ and gender-affirming healthcare.
**Older LGBTQ+ Canadians' Experiences of Prejudice and Discrimination Over the Life Course**	Hurd, Laura and Mahal, Raveena	Canada	2025	Using life-course perspectives to explore older LGBT+ Canadians experiences of prejudice and discrimination.	Semi-structured qualitative interviews	30	Three themes emerged: Traumatic interpersonal encounters in childhood and young adulthood, including discrimination, stigmatization, abuse, and healthcare bias; Institutional oppression in middle age,

							reflecting continued heteronormativity, cisgenderism, and barriers to culturally competent, gender-affirming care affecting work and life; and Later-life invisibilization, with participants feeling overlooked socially yet overexposed in healthcare. The study notes the need for further research.
**Geriatric Transgender Care**	Javier, Noelle M.	United States	2019	To highlight the needs off and barriers faced by older transgender patients.	Qualitative studies building on narratives	Not defined	Transgender and gender non-conforming older adults face barriers in healthcare, including discrimination, heteronormativity, and the need to educate caregivers. The chapter emphasizes the importance of providing compassionate, culturally aware, and inclusive care to support this population.
**Perceptions of ageing as an older gay man: a qualitative study**	Kushner, Bernie; Neville, Stephen and Adams, Jeffery	New Zealand	2013	To explore the ageing experiences of gay men in New Zealand over the age of 65 years.	Semi-structured interviews	12	Three themes emerged: Homophobia, describing experiences within families and internalized homophobia; Being with someone, highlighting the importance of later-life partnerships and loneliness due to age-related discrimination in the LGBT+ community; and Future care, reflecting fears of returning “to the closet,” desires for

							LGBT+–inclusive long-term care, and nondiscriminatory healthcare.
**Satisficing Death: Aging and End-of-Life Preparation Among Transgender Older Americans**	Lampe, Nik M.	United States	2024	To examine how transgender older adults percive and plan for aging and end-of-life experiences through advance care planning.	Semi-structured interviews	47	The study identified six overarching themes: (1) reevaluating a good death for trans elders, (2) medical mistreatment and neglect in older adult care settings, (3) lack of social support, (4) employing resourceful strategies to satisfice death, (5) formalizing advance care planning documents, and (6) selecting healthcare agents. The concept of “satisficing death” was introduced, referring to efforts to secure a “good death” within existing societal constraints. Participants reported concerns about medical mistreatment and neglect in older adult care settings. A lack of social support, particularly from family due to LGBTQIA+ stigma, was also described and was reported to influence advance care planning.<br>Participants described various strategies to approximate their preferred end-of-life outcomes. The majority had formalized advance care

							planning documents and had identified healthcare agents, with underlying considerations informing these decisions.
**"We grow older. We also have lots of sex. I just want a doctor who will at least talk about it.": Transgender, non-binary, and intersex older adults in sexual and reproductive healthcare.**	Lampe, Nik M and Pfeffer, Carla A	United States	2024	To examine how transgender, non-binary and intersex older adults experience and mitigate inequality in sexual and reproductive healthcare.	Semi-structured interviews	50	The study identified themes highlighting gaps in culturally competent care for older transgender, non-binary, and intersex adults: (1) sexual and reproductive healthcare competency gaps; (2) lack of sexual/gender minority affirmation and inclusion; (3) lack of older-age affirmation; (4) resourcefulness strategies to reduce inequalities; and (5) avoidance of services. Strengthening cultural competency in healthcare was recommended.
**"My support groups... have saved my life": facilitators of positive or satisfactory experiences in behavioral healthcare for transgender and gender nonconforming older adults.**	Lampe Nik M.; Rodill Zena E. and Nowakowski, Alexandra C.H.	United States	2024	To investigate facilitators contributing to positive or satisfactory behavioural healthcaer among older transgender in the United States	Semi-structured interviews	47	Three themes emerged for older transgender and gender nonconforming adults: (1) Engaging with compassionate, patient-centered behavioral healthcare, highlighting the value of knowledgeable providers with prior experience; (2) Accessing culturally tailored peer-support groups, facilitating community support and

							resource access; and (3) Equitable access to gender-affirming care and social services, improving mental health and healthcare experiences. Further research was recommended.
**Investigating the Needs and Concerns of Lesbian, Gay, Bisexual, and Transgender Older Adults: The Use, of Qualitative and Quantitative Methodolgy**	Orel, Nancy A.	United States	2014	To identify the common themes regarding th needs, concerns, and issues affecting a select group of older LGBT persons.	Mixed-method utilising a quatitative studies using LGBT Elders Needs Assesment Survey and qualitative studies, including focus groups and in-depth interviews	26	Focus groups with older LGB adults identified seven key areas of concern: medical/healthcare, legal, institutional/housing, spiritual, family, mental health, and social needs. Medical and healthcare needs were primary, with participants reporting discrimination, bias, and heteronormativity. Legal concerns focused on insufficient protections for same-sex partners.
**Coping strategies used by LGB older adults in facing and anticipating health challenges: A narrative analysis**	Seelman, Kristie L; Lewinson, Terri; Engleman, Lily; Maley, Olicia R. and Allen, Alex	United States	2017	Coping strategies used by LGB older adults in facing and anticipating health challenges: A narrative analysis	Qualitative narrative interviews	9	Two overarching themes were identified: (1) health challenges and (2) coping strategies.<br>Most participants reported experiencing significant health challenges, encompassing both acute health crises and long-term management of chronic comorbidities. These experiences were reflected in the subthemes "being

							stricken instantly" and "dealing with it over years." Participants described several coping strategies in response to these challenges, including engagement in health-promoting behaviours, shifting perspectives on health and the body, reliance on spirituality for comfort, and acceptance of the end of life.
**Motivations for advance care and end-of-life planning among lesbian, gay and bisexual older adults.**	Seelman, Kristie L; Lewinson, Terri; Engleman, Lily and Allen, Alex	United States	2019	The aim of the study was to look at motivations for advance care and end-of-life wishes for LGB older adults.	Mixed-method study using in-depth interviews as well as brief pen-and-paper survey.	9	Among older LGB adults, three themes motivated advance planning: (1) Desire for agency, ensuring control over caregiving and end-of-life decisions; (2) Learning from others, including professionals and loved ones; and (3) Reducing conflict, minimizing confusion for loved ones. The study emphasizes respecting older LGB adults' wishes.

Title	Was there a clear statement of the aims of the research?	Is a qualitative methodology appropriate?	Was the research design appropriate to address the aims of the research?	Was the recruitment strategy appropriate to the aims of the research?	Was the data collected in a way that addressed the research issue?	Has the relationship between researcher and participants been adequately considered?	Have ethical issues been taken into consideration?	Was the data analysis sufficiently rigorous?	Is there a clear statement of findings?	How valuable is the research?	Evaluation
**Adan 2021, Worry and Wisdom: A Qualitative Study of Transgender Elders' Perspectives on Aging.**	Yes	Yes	Can't tell	Yes	Yes	No	Yes	Yes	Yes	Yes	High
**Ansara 2015, Challenging cisgenderism in the ageing and aged care sector: Meeting the needs of older people of trans and/or non-binary experience.**	Yes	Yes	Can't tell	Can't tell	Can't tell	Can't tell	Can't tell	Yes	Yes	Yes	Moderat
**Burton 2020, "Things Are Different Now But"": Older LGBT Adults' Experiences and Unmet Needs in Health Care."**	Yes	Yes	Yes	Yes	Yes	Yes	Yes	Yes	Yes	Yes	High
**Butler 2017, Older lesbians' experiences with home care: Varying levels of disclosure and discrimination.**	Yes	Yes	Yes	Yes	Yes	Can't tell	Can't tell	Yes	Yes	Yes	Moderat
**Butler 2018, Older lesbians receiving home care: formal and informal dimensions of caregiving.**	Yes	Yes	Yes	Yes	Yes	Can't tell	Can't tell	Yes	Yes	Yes	High
**Goldhammer 2019, Meeting the Behavioral Health Needs of LGBT Older Adults.**	Yes	Yes	Yes	No	No	No	No	Yes	Yes	Yes	Moderat
**Hoekstra-Pijpers 2022, Experiences of older LGBT people ageing in place with care and support: A window on ordinary ageing environments, home-making practices and meeting activities**	Yes	Yes	Yes	Yes	Yes	Yes	Yes	Yes	Yes	Yes	High

**Hurd 2023, "I Want to Grow Older With Dignity"": Older LGBTQ+ Canadian Adults' Perceptions and Experiences of Aging"**	Yes	Yes	Yes	Yes	Yes	Can't tell	Yes	Yes	Yes	Yes	High
**Hurd 2025, Older LGBTQ+ Canadians' Experiences of Prejudice and Discrimination Over the Life Course**	Yes	Yes	Yes	Yes	Yes	Can't tell	Yes	Yes	Yes	Yes	High
**Javier 2019, Geriatric transgender care**	Yes	Yes	Yes	No	Yes	No	No	Yes	Yes	Yes	Moderat
**Kushner 2013, Perceptions of ageing as an older gay man: a qualitative study.**	Yes	Yes	Yes	Yes	Can't tell	Yes	Yes	Yes	Yes	Yes	High
**Lampe 2024, "Satisficing Death: Aging and End-of-Life Preparation Among Transgender Older Americans"**	Yes	Yes	Yes	Yes	Yes	Yes	Yes	Yes	Yes	Yes	High
**Lampe 2024, "We grow older. We also have lots of sex. I just want a doctor who will at least ask about it."": Transgender, non-binary, and intersex older adults in sexual and reproductive healthcare."**	Yes	Yes	Yes	Yes	Yes	Can't tell	Can't tell	Yes	Yes	Yes	Moderat
**Lampe 2024, "My support groups... have saved my life."": facilitators of positive or satisfactory experiences in behavioral healthcare for transgender and gender nonconforming older adults"**	Yes	Yes	Yes	Yes	Yes	Can't tell	Yes	Yes	Yes	Yes	High
**Orel 2014, "Investigating the needs and concerns of lesbian, gay, bisexual, and transgender older adults: the use of qualitative and quantitative methodology."**	Yes	Yes	Yes	No	Yes	No	Can't tell	Yes	Yes	Yes	Moderat

**Seelman 2017, Coping strategies used by LGB older adults in facing and anticipating health challenges: A narrative analysis**	Yes	Yes	Yes	Yes	Yes	Can’t tell	Yes	Yes	Yes	Yes	High
**Seelman 2019, "Motivations for advance care and end-of-life planning among lesbian, gay, and bisexual older adults."**	Yes	Yes	Yes	Yes	Yes	Can't tell	Can't tell	Yes	Yes	Yes	Moderat

**Title**	Did the systematic review address a clearly formulated research question?	Did the researchers search for appropriate study design(s) to answer the research question	Were all the relevant primary research studies likely to have been included in the systematic review? a) Searching for primary research studies	b) Screening primary research studies from the search	c) Selecting primary research studies to include in the systematic review	d) Summarising the search and its outputs	Did the researchers extract, and present information from the individual primary research studies appropriately and transparently? (a) Extraction of data	(b) Presentation of data
**Fasullo 2022,LGBTQ Older Adults in Long-Term Care Settings: An Integrative Review to Inform Best Practices.**	Yes	Yes	Yes	Yes	Yes	Yes	Yes	Yes

**Title**	**Did the researchers analyse the pooled results of the individual primary research studies appropriately?**	**6.1 Subgroup analysis**	**6.2 Meta-regression**	**Did the researchers report any limitations of the systematic review and, if so, do the limitations discussed cover all the issues you have identified during critical appraisal?**	**7.1 Subgroup analysis**	**7.2 Meta-regression**	**Would the benefits of acting upon the results outweigh any potential disadvantages, harms and/or additional demand for resources associated with acting on the results?**	**Can the results of the systematic review be applied to your local population/in your local setting or context?**	**If actioned, would the findings from the systematic review represent greater or additional value for the individuals or populations for whom you are responsible?**	**Evaluation**
**Fasullo 2022,LGBTQ Older Adults in Long-Term Care Settings: An Integrative Review to Inform Best Practices.**	Yes	Yes	No	Yes	No	No	Can’t tell	Yes	Yes	High
