# Supplementary material for: Healthcare Experiences of Older Adults with an LGBT+ Identity: An Integrative Review
Source: Healthcare (Basel). 2026 Apr 21;14(8):1110. doi: 10.3390/healthcare14081110 (PMC13115870; doi:10.3390/healthcare14081110)
Supplement: Supplementary file 1 [file healthcare-14-01110-s001.zip › Table S4 - CASP evaluation.pdf]

Title	Was there a clear statement of the aims of the research?	Is a qualitative methodology appropriate?	Was the research design appropriate to address the aims of the research?	Was the recruitment strategy appropriate to the aims of the research?	Was the data collected in a way that addressed the research issue?	Has the relationship between researcher and participants been adequately considered?	Have ethical issues been taken into consideration?	Was the data analysis sufficiently rigorous?	Is there a clear statement of findings?	How valuable is the research?	Evaluation
**Adan 2021, Worry and Wisdom: A Qualitative Study of Transgender Elders' Perspectives on Aging.**	Yes	Yes	Can't tell	Yes	Yes	No	Yes	Yes	Yes	Yes	High
**Ansara 2015, Challenging cisgenderism in the ageing and aged care sector: Meeting the needs of older people of trans and/or non-binary experience.**	Yes	Yes	Can't tell	Can't tell	Can't tell	Can't tell	Can't tell	Yes	Yes	Yes	Moderat
**Burton 2020, "Things Are Different Now But"": Older LGBT Adults' Experiences and Unmet Needs in Health Care."**	Yes	Yes	Yes	Yes	Yes	Yes	Yes	Yes	Yes	Yes	High
**Butler 2017, Older lesbians' experiences with home care: Varying levels of disclosure and discrimination.**	Yes	Yes	Yes	Yes	Yes	Can't tell	Can't tell	Yes	Yes	Yes	Moderat
**Butler 2018, Older lesbians receiving home care: formal and informal dimensions of caregiving.**	Yes	Yes	Yes	Yes	Yes	Can't tell	Can't tell	Yes	Yes	Yes	High
**Goldhammer 2019, Meeting the Behavioral Health Needs of LGBT Older Adults.**	Yes	Yes	Yes	No	No	No	No	Yes	Yes	Yes	Moderat
**Hoekstra-Pijpers 2022, Experiences of older LGBT people ageing in place with care and support: A window on ordinary ageing environments, home-making practices and meeting activities**	Yes	Yes	Yes	Yes	Yes	Yes	Yes	Yes	Yes	Yes	High

**Hurd 2023, "I Want to Grow Older With Dignity"": Older LGBTQ+ Canadian Adults' Perceptions and Experiences of Aging"**	Yes	Yes	Yes	Yes	Yes	Can't tell	Yes	Yes	Yes	Yes	High
**Hurd 2025, Older LGBTQ+ Canadians' Experiences of Prejudice and Discrimination Over the Life Course**	Yes	Yes	Yes	Yes	Yes	Can't tell	Yes	Yes	Yes	Yes	High
**Javier 2019, Geriatric transgender care**	Yes	Yes	Yes	No	Yes	No	No	Yes	Yes	Yes	Moderat
**Kushner 2013, Perceptions of ageing as an older gay man: a qualitative study.**	Yes	Yes	Yes	Yes	Can't tell	Yes	Yes	Yes	Yes	Yes	High
**Lampe 2024, "Satisficing Death: Aging and End-of-Life Preparation Among Transgender Older Americans"**	Yes	Yes	Yes	Yes	Yes	Yes	Yes	Yes	Yes	Yes	High
**Lampe 2024, "We grow older. We also have lots of sex. I just want a doctor who will at least ask about it."": Transgender, non-binary, and intersex older adults in sexual and reproductive healthcare."**	Yes	Yes	Yes	Yes	Yes	Can't tell	Can't tell	Yes	Yes	Yes	Moderat
**Lampe 2024, "My support groups... have saved my life."": facilitators of positive or satisfactory experiences in behavioral healthcare for transgender and gender nonconforming older adults"**	Yes	Yes	Yes	Yes	Yes	Can't tell	Yes	Yes	Yes	Yes	High
**Orel 2014, "Investigating the needs and concerns of lesbian, gay, bisexual, and transgender older adults: the use of qualitative and quantitative methodology."**	Yes	Yes	Yes	No	Yes	No	Can't tell	Yes	Yes	Yes	Moderat

**Seelman 2017, Coping strategies used by LGB older adults in facing and anticipating health challenges: A narrative analysis**	Yes	Yes	Yes	Yes	Yes	Can’t tell	Yes	Yes	Yes	Yes	High
**Seelman 2019, "Motivations for advance care and end-of-life planning among lesbian, gay, and bisexual older adults."**	Yes	Yes	Yes	Yes	Yes	Can't tell	Can't tell	Yes	Yes	Yes	Moderat

**Title**	Did the systematic review address a clearly formulated research question?	Did the researchers search for appropriate study design(s) to answer the research question	Were all the relevant primary research studies likely to have been included in the systematic review? a) Searching for primary research studies	b) Screening primary research studies from the search	c) Selecting primary research studies to include in the systematic review	d) Summarising the search and its outputs	Did the researchers extract, and present information from the individual primary research studies appropriately and transparently? (a) Extraction of data	(b) Presentation of data
**Fasullo 2022,LGBTQ Older Adults in Long-Term Care Settings: An Integrative Review to Inform Best Practices.**	Yes	Yes	Yes	Yes	Yes	Yes	Yes	Yes

**Title**	**Did the researchers analyse the pooled results of the individual primary research studies appropriately?**	**6.1 Subgroup analysis**	**6.2 Meta-regression**	**Did the researchers report any limitations of the systematic review and, if so, do the limitations discussed cover all the issues you have identified during critical appraisal?**	**7.1 Subgroup analysis**	**7.2 Meta-regression**	**Would the benefits of acting upon the results outweigh any potential disadvantages, harms and/or additional demand for resources associated with acting on the results?**	**Can the results of the systematic review be applied to your local population/in your local setting or context?**	**If actioned, would the findings from the systematic review represent greater or additional value for the individuals or populations for whom you are responsible?**	**Evaluation**
**Fasullo 2022,LGBTQ Older Adults in Long-Term Care Settings: An Integrative Review to Inform Best Practices.**	Yes	Yes	No	Yes	No	No	Can’t tell	Yes	Yes	High
